# A mini-review of Vutrisiran and Eplontersen in hereditary transthyretin-mediated amyloidosis with polyneuropathy

**DOI:** 10.1097/MD.0000000000038767

**Published:** 2024-06-28

**Authors:** Gbolahan Olatunji, Emmanuel Kokori, Israel Charles Abraham, Oluwatobi Omoworare, Doyin Olatunji, Chimezirim Ezeano, Babawale Emmanuel Adeoba, Anthony Chidera Stanley, Awoyinfa Michael Oluwatobiloba, Omidiran Basit Oluwademilade, Kale Mekoya Shimelis, Olawale Olanisa, Nicholas Aderinto

**Affiliations:** a Department of Medicine and Surgery, University of Ilorin, Ilorin, Nigeria; b Department of Medicine and Surgery, Lagos State University College of Medicine, Lagos, Nigeria; c Department of Health Sciences, Western Illinois University, Macomb, IL; d Department of Health, University of North Texas, Health Science Centre, Fort Worth, TX; e Department of Medicine and Surgery, Griffith College Dublin, Dublin, Ireland; f Department of Medicine and Surgery, University of Calabar, Calabar, Nigeria; g College of Medicine, University of Lagos, Lagos, Nigeria; h Department of Medicine and Surgery, Afe Babalola University, Ado-Ekiti, Nigeria; i Department of Medicine, Addis Ababa University, Addis Ababa, Ethiopia; j Department of Internal Medicine, Trinity Health Centre, Grand Rapids, MI; k Department of Medicine and Surgery, Ladoke Akintola University of Technology, Ogbomoso, Nigeria.

**Keywords:** ATTRv amyloidosis polyneuropathy, Eplontersen, hereditary transthyretin-mediated amyloidosis, Vutrisiran

## Abstract

Hereditary transthyretin-mediated amyloidosis (ATTRv amyloidosis), known as Corino de Andrade disease, is a rare neurodegenerative disorder with a significant global impact characterized by the misfolding of transthyretin (TTR) protein leading to amyloid aggregation, ATTRv amyloidosis, especially with polyneuropathy, poses a considerable challenge in managing its rapid progression and debilitating effects. This mini-review focuses on the recent advancements in the treatment landscape for ATTRv amyloidosis with polyneuropathy, specifically the RNA interference therapeutic Vutrisiran and the ligand-conjugated antisense oligonucleotide Eplontersen. We aim to provide a comprehensive overview of the mechanisms, current evidence from clinical trials, and future directions for these novel therapeutic agents. Vutrisiran and Eplontersen have demonstrated significant clinical efficacy in improving neuropathic impairment, quality of life, and serum TTR levels in various trials. The distinct mechanistic approaches of these therapies, coupled with their acceptable safety profiles, offer promising avenues for addressing the complexities of ATTRv amyloidosis with polyneuropathy. The introduction of Vutrisiran and Eplontersen marks a pivotal moment in the quest for effective therapies against ATTRv amyloidosis with polyneuropathy. While clinical evidence is promising, ongoing research is crucial to deepen mechanistic understanding and address research gaps. Future perspectives include the potential expansion of therapeutic options and a more inclusive approach to cater to the diverse needs of individuals globally. This mini-review provides valuable insights into the evolving landscape of ATTRv amyloidosis management and sets the stage for further exploration in this challenging domain.

## 1. Introduction

Hereditary transthyretin-mediated amyloidosis (ATTRv amyloidosis), commonly referred to as Corino de Andrade disease, is a rare neurodegenerative disorder acquired through an autosomal dominant inheritance pattern.^[1]^ ATTRv amyloidosis affects approximately 50,000 people worldwide.^[1]^ This debilitating disease is marked by its aggressive nature, causing rapid progressive deterioration of vital bodily functions.^[2]^ ATTRv amyloidosis is linked to the misfolding of the transthyretin (TTR) protein, resulting in its aggregation into an insoluble amyloid form resistant to proteolysis.^[3,4]^ The subset of individuals with ATTRv amyloidosis with polyneuropathy is estimated at around 10,000 globally. However, this figure may underestimate the prevalence due to a reduction in diagnoses associated with nonspecific clinical features common to other diseases.^[2]^

ATTRv amyloidosis with polyneuropathy is characterized by a rapid and degenerative progression, significantly impacting the overall well-being of individuals by hindering their ability to perform daily tasks.^[5]^ Motor function is compromised, and various sensory abnormalities are prevalent, including numbness, pain, tingling, and burning sensations.^[6]^ Early and effective therapies targeting the pathophysiologic process underlying the condition are crucial in managing multiple organ dysfunction, limiting the disease burden and progression.^[7]^ Strategies include silencing the TTR gene to limit its natural production and stabilizing the tetrameric structure of the TTR protein to reduce misfolding and the formation of nonprotein aggregates.^[8,9]^

The limited therapeutic options have shown the need for innovative and targeted interventions to address the underlying pathophysiology and alleviate the burden of this devastating disorder. Gene-silencing therapies for ATTRv amyloidosis with polyneuropathy have evolved, with Patisiran and Inotersen being early advancements.^[10]^ These paved the way for Vutrisiran and Eplontersen, the latest gene-silencing medications for ATTRv amyloidosis with polyneuropathy.^[11]^ Before gene therapies, treatment options included liver transplantation and TTR stabilizers.^[10]^ Notably, diflunisal and tafamidis were explored as TTR kinetic stabilizers, altering the natural history of ATTRv amyloidosis with polyneuropathy.^[10]^

The recent approval of Vutrisiran and Eplontersen marks significant milestones in the pursuit of effective therapies for ATTRv amyloidosis with polyneuropathy.^[1,2]^ Vutrisiran is an RNA interference (RNAi) therapeutic that reduces serum TTR levels by inhibiting the synthesis of variant and wild-type TTR.^[1]^ Similarly, Eplontersen is a N-acetyl galactosamine (GalNAc)-conjugated antisense oligonucleotide (ASO) that preferentially targets hepatic TTR mRNA, thereby inhibiting TTR protein synthesis.^[2]^ This paper aims to review the available evidence regarding using Vutrisiran and Eplontersen in treating ATTRv amyloidosis with polyneuropathy.

## 2. Mechanisms and targets

ATTRv amyloidosis with polyneuropathy represents a variable clinical presentation characterized by abnormal polysystemic extracellular amyloid deposition, disrupting the physiology of tissues and organs.^[7]^ Amyloidosis, whether local or systemic, results from the abnormal deposition of pernicious protein formations – beta-sheet fibrillar aggregates that accumulate in different organs, causing disruptions in normal function and gross morphology.^[8]^ These aggregates in organs such as the kidneys, vessels, heart, spleen, liver, and nerves contribute to various clinical presentations and syndromes, including cardiomegaly, hepatomegaly, renal dysfunction, visual disturbances, autonomic dysfunction, and neuropathy.^[12]^ The disease is heterogeneously inherited and can also be acquired, with classifications based on etiologies and chemical constituents, including AA, AL, ATTR, and dialysis-related amyloidosis.^[8]^

TTR, a highly conserved protein, is crucial in transporting thyroxine and retinol-binding protein, with evidence supporting its involvement in neuroprotection and neural regeneration in response to injuries.^[12]^ Hereditary TTR amyloidosis (ATTRv amyloidosis) is a rare and mortal autosomal dominant genetic gain-of-function disorder caused by mutations in the TTR gene.^[13]^ This mutation results in misfolded monomers of TTR that accumulate to form amyloid fibrils, leading to multisystem involvement with a stronger predilection for the heart and nerves, causing cardiomyopathy and polyneuropathy, usually manifesting in midlife.^[14]^

Vutrisiran, belonging to the class of double-stranded siRNA-GalNAc conjugates, serves as a gene silencer.^[13]^ It dissolves abnormal TTR mRNA by binding and silencing the messenger RNA that encodes disease-causing TTR. Vutrisiran targets both mutant and wild-type TTR mRNA, disrupting the transfer of codes from the DNA in the nucleus to the sites of ribosomal protein synthesis. Consequently, this results in a reduction of serum TTR protein levels.^[13]^ Eplontersen, a self-administered subcutaneous medication, falls into the class of ligand-conjugated antisense oligonucleotides (LICA).^[2]^ ASOs are synthetic, single-stranded oligonucleotides designed to bind to RNAs.^[15]^ Like Vutrisiran, Eplontersen acts as a gene silencer inhibiting nuclear-encoded protein synthesis. However, its mechanism involves ligands conjugated with ASOs.^[16]^ These ligands exhibit a high affinity for specific molecules/receptors, ensuring targeted tissue delivery. Eplontersen demonstrates increased potency and receptor-mediated hepatocyte uptake compared to unconjugated ASOs (Inotersen).^[14]^ ASOs accumulate in tissues like the kidneys, but ligand conjugation, as seen in Eplontersen, ensures targeted delivery.^[17]^ ASO binds to TTR mRNA without interfering with its antisense activity, ultimately causing RNA cleavage or blockage and altering cellular synthesis, reducing circulating TTR.^[18]^

## 3. Current evidence

Masri et al^[16]^ conducted an analytical observational study within the neuropathy TTR amyloidosis study (NEURO-TTRansform) trial involving 168 patients with ATTRv amyloidosis with polyneuropathy. The Eplontersen group, comprising 144 patients, showed a positive correlation between Eplontersen therapy and improved cardiac structure and function in the cardiomyopathy subgroup. Notably, adverse events were consistent with those observed in the NEURO-TTransform trial, with no specific negative outcomes reported. Similarly, in phase 3, open-label, single-group, multicenter, international, randomized trial (NEURO-TTransform trial), Coelho et al^[2]^ included 168 patients, with 144 in the Eplontersen group. Over 65 weeks, the Eplontersen group exhibited improvements in neuropathic impairment, quality of life, and serum TTR levels. Significant reductions in the modified Neuropathy Impairment Score + 7 (mNIS + 7), Norfolk Quality of Life-Diabetic Neuropathy (Norfolk QoL-DN) total score, and serum TTR levels were observed, with no negative outcomes leading to intervention cessation. However, 6 patients (4%) in the Eplontersen group withdrew, and 2 deaths occurred during treatment.

Adams et al^[1]^ conducted a phase 3, open-label, international, randomized trial (HELIOS-A trial) with 164 patients. Vutrisiran met the primary endpoint of change from baseline in modified Neuropathy Impairment Score + 7 (mNIS + 7) at 9 months (*P* = 3.54 × 10^−12^), and all secondary efficacy endpoints; significant improvements versus external placebo were observed in Norfolk QoL-DN, 10-meter walk test (both at 9 and 18 months), mNIS + 7, modified body-mass index, and Rasch-built Overall Disability Scale (all at 18 months). TTR reduction with Vutrisiran Q3M was noninferior to within-study Patisiran Q3W. Most adverse events were mild or moderate in severity and consistent with ATTRv amyloidosis natural history. There were no drug-related discontinuations or deaths. Moreover, in phase 1, a single ascending dose clinical trial by Habtemariam et al^[18]^ involving 80 healthy subjects, Vutrisiran treatment achieved potent and sustained TTR reduction in a dose-dependent manner, with a mean maximum TTR reduction of 57% to 97%, maintained for ≥90 days post-dose. Vutrisiran was rapidly absorbed (peak plasma concentration 3–5 hours post-dose), had a short plasma half-life (4.2–7.5 hours), and plasma concentrations increased dose-proportionally. Pharmacodynamic and pharmacokinetic results were similar in Japanese and non-Japanese subjects. Vutrisiran had an acceptable safety profile; the most common treatment-related adverse event was mild, transient injection site reactions in 4 (6.7%) Vutrisiran-treated subjects.

## 4. Future directions and perspectives

The recent approval of Vutrisiran and Eplontersen has ushered in a new era in managing ATTRv amyloidosis with polyneuropathy. While the mechanisms of Vutrisiran and Eplontersen are well-established, ongoing studies should explore the interactions within the cellular milieu. Beyond understanding gene-silencing, investigating the specific cascades, signal transductions, and molecular interactions can contribute to a better grasp of their mode of action.

The existing evidence has provided valuable insights into the short to mid-term safety and efficacy of Vutrisiran and Eplontersen. However, longitudinal studies with extended follow-up periods are important. Investigating the long-term impact on disease progression, organ function, and the emergence of any latent adverse effects will be pivotal in establishing the enduring safety and efficacy profiles of these interventions.

The current landscape hints at the potential benefits of combining therapeutic approaches in managing ATTRv amyloidosis. Future studies should explore synergies between Vutrisiran, Eplontersen, and other emerging therapies like acoramidis (AG10), which demonstrated a greater degree of TTR stabilization in patients with TTR amyloid cardiomyopathy in a recently concluded phase 3 randomized controlled trial.^[19]^ At the time of this publication, no studies are exploring the synergistic effects that might exist between acoramidis in combination with Vutrisiran and Eplontersen in treating ATTRv amyloidosis. Given the similar underlying pathology of TTR destabilization and dissociation in ATTRv amyloidosis and ATTRv amyloidosis with cardiomyopathy, exploring the potential benefits of acoramidis in combination with Vutrisiran and/or Eplontersen in ATTRv amyloidosis therapy might reveal scientific insights into managing this debilitating condition. Furthermore, seeking to understand the potential synergistic effects that may lie in the combination of Vutrisiran and/or Eplontersen in combination with older TTR stabilizers like Diflusinal and Tafamidis in the treatment of ATTRv amyloidosis might aid the discovery of combination regimens that might cater to different variants of the disease. The authors recognize the valuable contributions of previous clinical trials in the management of ATTRv amyloidosis and also appreciate that conducting clinical trials is difficult for a disease of such rarity; nevertheless, a more refined therapeutic approach can be achieved while the benefits of currently available therapies are maximized for patient care. Exploring these combinatorial strategies might offer enhanced efficacy and better tolerability, potentially addressing the disease heterogeneity observed in patients with ATTRv amyloidosis. In addition, the rarity of ATTRv amyloidosis and its diverse manifestations shows the need for personalized treatment strategies. Genetic, phenotypic, and environmental factors may influence the response to Vutrisiran and Eplontersen. Future research should focus on identifying biomarkers to predict treatment response, allowing clinicians to tailor interventions to individual patient profiles.

Combining regions with a few patients for statistical purposes may introduce bias. Future studies should prioritize diverse and representative participant inclusion, considering geographic, genetic, and phenotypic variations. This will contribute to a better understanding of the impact of Vutrisiran and Eplontersen across diverse populations.

## 5. Conclusion

The advent of Vutrisiran and Eplontersen represents a significant stride in pursuing effective therapeutic options for ATTRv amyloidosis with polyneuropathy. The limited therapeutic option for this debilitating condition has been met with innovative solutions that target the fundamental processes driving the disease. The evidence gleaned from clinical trials, such as the NEURO-TTRansform and HELIOS-A trials, underscores the clinical efficacy of these novel treatments. Vutrisiran and Eplontersen have exhibited positive outcomes in improving neuropathic impairment, quality of life, and serum TTR levels. Importantly, their safety profiles, as evidenced by mild or moderate adverse events, showcase their viability as therapeutic options.

Vutrisiran, operating as an RNAi therapeutic, and Eplontersen, utilizing ligand-conjugated ASOs, showcase distinct mechanistic approaches. Vutrisiran ability to target mutant and wild-type TTR mRNA highlights its broad-spectrum effectiveness, while Eplontersen ligand conjugation enhances specificity and tissue-targeting capabilities. Despite these advancements, our exploration underscores the imperative need for further research. Advancing mechanistic understanding is critical for optimizing the application of Vutrisiran and Eplontersen. The interactions within the cellular milieu, tissue-specific responses, and potential side effects necessitate continued scrutiny for a more refined therapeutic approach.

Several research gaps and unmet needs persist, accentuating the complexity of managing ATTRv amyloidosis. Limitations such as small sample sizes, geographical biases, and the impact of external factors like the COVID-19 pandemic call for a concerted effort to address these gaps. Future studies should prioritize larger, diverse cohorts and consider the influence of external variables on treatment outcomes. Looking ahead, the future of ATTRv amyloidosis management appears promising. The ongoing CARDIO-TTRANSform trial and the Phase 3 clinical trial evaluating the effectiveness and safety of Vutrisiran present opportunities for expanding therapeutic potential. Also, a phase 1 study is underway to appraise the safety, tolerability, pharmacokinetics and pharmacodynamics of NTLA-2001 in patients with ATTRv amyloidosis with polyneuropathy and in patients with ATTR amyloidosis with cardiomyopathy. The EDONA trial is also presently seeking to develop methods that will aid in the early identification of neuropathy in patients with ATTRv amyloidosis, and an observational study aiming to identify the first signs of clinically significant organ involvement in patients with ATTRv amyloidosis is in its enrollment phase at this time. The number of current and forthcoming studies underscore the promising future in managing ATTRv amyloidosis and other disease variants. Nevertheless, in regions with limited representation in current studies, the need for a global and inclusive approach remains unfulfilled. Studies currently in their recruitment phase should take note of this and assemble a more inclusive patient demography.

## Author contributions

**Conceptualization:** Gbolahan Olatunji, Nicholas Aderinto.

**Writing – original draft:** Gbolahan Olatunji, Emmanuel Kokori, Israel Charles Abraham, Oluwatobi Omoworare, Doyin Olatunji, Chimezirim Ezeano, Babawale Emmanuel Adeoba, Anthony Chidera Stanley, Awoyinfa Michael Oluwatobiloba, Omidiran Basit Oluwademilade, Kaleb Mekoya Shimelis, Olawale Olanisa, Nicholas Aderinto.

**Writing – review & editing:** Gbolahan Olatunji, Emmanuel Kokori, Israel Charles Abraham, Oluwatobi Omoworare, Doyin Olatunji, Chimezirim Ezeano, Babawale Emmanuel Adeoba, Anthony Chidera Stanley, Awoyinfa Michael Oluwatobiloba, Omidiran Basit Oluwademilade, Kaleb Mekoya Shimelis, Olawale Olanisa, Nicholas Aderinto.
